# Editorial: Social determinants of health for the global aging population in pandemic and disaster environments

**DOI:** 10.3389/fpubh.2025.1552408

**Published:** 2025-02-06

**Authors:** Lené Levy-Storms, Soohyoung Rain Lee, Camille Castelyn, Mary Beth Morrissey, Marcia G. Ory

**Affiliations:** ^1^Department of Social Welfare, Luskin School of Public Affairs, UCLA, Los Angeles, CA, United States; ^2^Wurzweiler School of Social Work, Yeshiva University, New York City, NY, United States; ^3^Albert Luthuli Leadership Institute, University of Pretoria, Pretoria, Gauteng, South Africa; ^4^School of Public Health, Texas A&M University, College Station, TX, United States

**Keywords:** social determinants, health, global aging, disasters, COVID pandemic, systems approach, complexity

This Research Topic offers insights into various social determinants of health among older adults across the world, particularly in the context of pandemics and disaster environments. In this Research Topic, health is defined broadly, capturing physical, psychological, literacy, and healthcare services domains. Studies address health issues at individual, organizational, systems, and country levels using various research designs, including qualitative, quantitative, mixed methods, and systematic reviews. Locations pertain to both countries and continents, including the U.S., Europe (i.e., Spain and Ireland), Australia, Africa, the Middle East (i.e., Israel), and Asia (i.e., Hong Kong). The substantive scope of social issues, health, levels of study, research designs, and varied international locations speak to the relevance of pandemics and disasters to everyone, everywhere. The focus on older adults calls attention to the unique vulnerabilities of the global aging population.

The following is a summary of each of the 12 accepted articles, their research focus, and general relevance to the public health and aging field.

Castelyn's commentary leads this special topic with a call for leadership from a systems approach, which differs from a more conventional people-oriented approach. Moreover, she points to the benefits of a crisis leadership systems approach, especially when dealing with “wicked problems” or complex and messy scenarios where there is no obvious solution available, much like what the world has been dealing with from COVID-19. The crisis leadership systems approach goes beyond any one organization's boundaries to encompass multiple larger healthcare systems. This is what Castelyn refers to as a leader-centric approach since leaders from different levels and places cross boundaries and underscores that in crises this leadership takes a proactive stance to prepare for how to resolve such “wicked problems.” She closes with six practical steps to achieve a systems leadership approach that incorporates “space” for paradoxes surrounding power, uncertainty, and conflict noting how these issues contribute to, not threaten, substantive action and progress. This systems leadership approach holds much promise for effectively providing necessary medical care to “forgotten” populations, especially older adults.

In the first research article, Wilbur et al. address the exacerbated vulnerability of having a disability, aging, or caregiving during COVID-19 in low- and middle-income countries (LMICs) through an observational study of the Hygiene Behavior Change Coalition (HBCC). Based on using a water, sanitation, and hygiene (WASH) checklist applied to 137 documents from HBCC grantees' interventions, the results indicated that having a disability, aging, and caregiving targets occurred but interventions did not ensure their participation in WASH infrastructure. The authors recommend more explicit targets with monitored actions to ensure benefits from intervention efforts and honor such vulnerable populations' inclusivity rights.

Williams et al. explored the effectiveness of targeted messaging around “self-isolation” and “social distancing” in Australia using an online survey of 3,300 adults aged 18+ through 80+. Analyses focused on comparisons across age in groups of 10 years. Results indicated that age differences existed in the correct understanding of messaging on these preventive behaviors as well as in the source of the messaging with older adults being more likely to get information from TV compared to younger adults. Regardless of age groups, messages were confusing and often perceived incorrectly with no clear pattern of enhanced understanding by either older or younger adults. Most concerning, though, were the findings that older adults may not have received needed medical attention because they misunderstood protocols to stay isolated and/or maintain social distancing.

Guzman et al. employed qualitative methods to explore 57 older adults' perceptions of how individual, social, and environmental factors intersected with their health and well-being during COVID-19 in 2021 from the Wellbeing, Interventions, and Support during Epidemics (WISE) study. Community-dwelling participants living in Ireland varied in their concerns, capabilities, and roles in society. Findings underscored how a one-size-fits-all approach based on chronological age did not suit them well. Implications include the need for adaptive strategies for the development of age-friendly interventions during such crises.

Using a mixed-method study design, Yang et al. focused on 109 home-based and community care services staff members in Hong Kong to implement and evaluate a risk management process and service enhancement for home- and community-based services in 2021 and 2022 during COVID-19. Both quantitative and qualitative results suggest that staff members' regular training, updated guidelines, and proactive phone calls to older adults all helped the quality of the services. Implications include the value of combining standard protocols with outreach efforts for community social services in general and especially during disasters.

Shaked et al. investigated how social and medical factors affected medical services use among 102,303 older adults during two periods in 2019 during the COVID-19 lockdown in Israel. Findings revealed how social factors strongly predicted reduced medical services use during both periods but medical needs were also reduced for those older adults with social supports. Implications suggest that older adults living in the community fare relatively much better—even well—when they have access to social support. Thus, governmental organizations need to allocate ample financial resources to ensure social support and services for vulnerable populations.

Wang et al. used the Survey of Health, Aging, and Retirement in Europe (SHARE) and Israel in 2017 and two rounds during COVID in 2020 and 2021 to understand how psychological well-being is associated with hospitalizations and mortality from COVID. Based on 3,886 adults aged 50 years and older, findings indicate that lower psychological well-being independently increased the risks for hospitalization and mortality. Implications point to psychological risk factors for poor physical outcomes and suggest the need for further research and intervention.

Cases et al. also examined the risk for mortality among 175,497 older adults in long-term care institutions living in Catalonia during the period 2015 to 2022 using healthcare registries. A key aspect of the retrospective, observational study design included comparative analyses of pre-pandemic mortality with those deaths after COVID-19 began. Findings indicate excess relative mortality for older adults during all waves, especially the first with additional nuanced findings for crude compared to standardized mortality rates within this population-based study of those over 50 years of age. Implications point toward the importance of using relative mortality measures in such vulnerable settings.

Nicklett et al. delved into the particular health issue of food insecurity over time and COVID-19 among a sample of 2,413 older adults from 2018 through 2020 from the Health and Retirement Survey (HRS). Food insecurity in this study entailed measuring having enough money to buy food. Findings included a doubling of food insecurity in this time period. In addition, a lower risk for food insecurity occurred among higher-income and better-educated individuals but a greater risk occurred for Black and rural individuals. Additional factors that increased the risk for food insecurity in 2020 included being younger, living with a disability, and renting. Implications point to the need for policies addressing the disparities in vulnerability to food insecurity especially during disaster periods.

Kibe et al. also studied food insecurity but using multiple measures of food quantity relative to need in households. The authors also measured the food environment and related it and food insecurity to overall diet quality among 102 older African American adults in Los Angeles, CA. Food insecurity but not food environment was related to dietary quality as well as recommended fruit and vegetable intake. Implications point to the dire need for intervention in this vulnerable population of underserved older African American adults.

Soo Oh et al. examined post-acute care (PAC) utilization among 4,310 Nevadans living with Alzheimer's disease and related disorders (ADRD) with extremity fractures after hospitalization pre- and post-COVID (i.e., 2018–19 and 2020–2021, respectively). They studied two rehabilitation locations as outcomes, both institution- and home-based, and analyzed predictors, including age, gender, race, fracture location, comorbidity, rural location, and pay source. Findings indicated that Hispanic populations had lower utilization rates of rehabilitation facilities and care at home. Overall, utilization rates shifted from institution- to home-based care, which, in turn, increased the risk of the disability and caregiver burden. Implications suggested the need for more geriatric healthcare workforce education to target underserved communities.

Lai et al. conducted a qualitative inquiry into the perception of heatwaves, vulnerabilities, and preparedness among older adults and service providers in Hong Kong. Semi-structured interviews included 46 older adults, 18 staff, and 2 district councilors. Findings indicated that older adults perceived increasingly hot weather but did not feel vulnerable. Staff and councilors described a lack of services in the community and education about heat threats to health. Implications point to the urgent need to take a systems approach to co-create a heat preparedness plan, improve community awareness, and buttress resources for protection especially for vulnerable older adults

In conclusion, the co-editors and I wish to thank all the authors, the reviewers, and the editorial board members for contributing to this Research Topic. Social determinants of health challenge healthcare systems based on their inherent complexity and require a coordinated effort across multiple global sectors. In editing this Research Topic, the authors call for further research, innovation, and critical thinking to learn from our past and prepare for our future.

